# A rare case of fatal cerebral phaeohyphomycosis caused by *Cladophialophora bantiana* in an immunocompetent individual in India

**DOI:** 10.18502/CMM.6.3.4498

**Published:** 2020-09

**Authors:** Ranjana Rohilla, Suneeta Meena, Nishant Goyal, Neelam Kaistha

**Affiliations:** 1 Department of Microbiology, All India Institute of Medical Science, Rishikesh, Uttarakhand, India; 2 Department of Laboratory Medicine, AIIMS, Delh; 3 Department of Neurosurgery, All India Institute of Medical Science, Rishikesh, Uttarakhand, India

**Keywords:** Brain abscess, Cerebral pheohyphomycosis, *Cladophialophora bantiana*, Melanised fungi

## Abstract

**Background and Purpose:**

Herein, we describe a rare case of fatal cerebral phaeohyphomycosis by *Cladophialophora bantiana* in an immunocompetent individual without any underlying risk factors.

**Case report:**

A 55-year-old female presented with a short history of fever for 1 month, as well as headache, sudden onset of right-sided upper and lower limb weakness, and loss of speech for 10 days. Contrast-enhanced magnetic resonance imaging scan revealed large, peripherally enhancing, well-defined, cystic, space- occupying, axial lesion in the left parietal lobe with a mass effect. The patient was subjected to craniotomy, and the drained pus revealed pigmented septate hyphae in potassium hydroxide mount examination, which was identified as *Cladophialophora bantiana*. The patient was started on amphotericin B and voriconazole. However, she developed acute respiratory infection leading to multi-organ failure and death on day 27 post-operation.

**Conclusion:**

In the absence of comparative trials owing to the rarity of the disease, the radical resection of lesion, drainage of pus, and prolonged targeted antifungal therapy with close postoperative radiological surveillance are the therapeutic measures of choice for patients with brain abscess caused by phaeoid fungi.

## Introduction

Central nervous system (CNS) infections caused by filamentous fungi, especially brown-black or dematiaceous fungi, are exceptionally rare conditions. The highly virulent fungi implicated in causing brain abscesses include *Cladophialophora bantiana, Rhinocladiella mackenziei, Verruconis gallopavum, Wangiella dermatitidis, Fonsecaea monophora, Lomentospora prolificans, Chaetomium strumarium, Exophiala dermatitidis, Bipolaris spicifera,* and *Curvularia lunata* [ 1 ]. These infections are rare and fatal and can affect both immunocompetent and immunocompromised patients. Moreover, they are often intractable to standard antifungal treatment regimens and require combined surgical and medical therapies [ 2 ].

A review of 101 cases of CNS phaeohy- phomycosis by Revankar et al. revealed that more than half of the patients were immunocompetent without any underlying diseases or risk factors. In the rest of the cases, the underlying risk factors included some degrees of immune dysfunction, malignancy (both hematologic malignancy and solid tumors), neutropenia, recent chemotherapy, bone marrow, and solid organ transplantation. Other less commonly associated risk factors were HIV with AIDS, corticosteroid use, trauma, and intravenous drug abuse [ 3 , 4 ].

While it is comparatively easy to diagnose and treat common fungal infections, such as *Candida* and Aspergillus, there is no specific diagnostic algorithm for the identification of rare dematiaceous fungi. *Cladophialophora bantiana* is the most common cause of brain abscess or cerebral pheohyphomycosis mostly in immunocompetent individuals, as well as disseminated systemic pheohyphomycosis among immunosuppressed patients [ 5 ]. The mortality rate due to cerebral phaeohyphomycosis can be as high as 70% if no medical or surgical intervention is planned. A successful treatment depends on radical neurosurgical resection, accurate microbiological identification of the fungi, and initiation of propitious personalized and targeted antifungal treatment [ 6 ].

Herein, we describe a case of fatal *C. bantiana* brain abscess in an immunocompetent individual from a small village in the Northern part of India.

## Case report

A 55-year-old female, who was a resident of a small village in the foothills of the Himalayas, presented to the emergency department of a tertiary healthcare institute in the State of Uttarakhand, India, with the chief complaints of fever for 1 month, headache, sudden onset of right-sided upper and lower limb weakness, and loss of speech for 10 days, as well as urinary incontinence for 7 days. Fever was mild to moderate and was not associated with any diurnal variation, chills and rigors, or rashes. It was associated with headache and 5-6 episodes of non- projectile vomiting. There was no history of breathing difficulties, burning micturition, seizures, or a history of weight loss. Additionally, there was no personal or family history of diabetes mellitus, hypertension, or tuberculosis. The systemic examination at the time of admission was indicative of a Glasgow coma scale (GCS) score of 15/15 (E4V5M6), blood pressure of 100/80 mm Hg, pulse rate of 110 per minute, and temperature of 98.4℉ with pallor.

On CNS examination, she was conscious and well oriented to time, place, and person; however, left-sided facial deviation was present. Plantar reflex was extensor on the right side, and deep tendon reflexes were exaggerated on the right knee and ankle. The grading of muscle power was 1/5 in the right-sided upper and lower limb. Respiratory system and abdominal examinations were within the normal limits. Meropenem (1 g, intravenous ), injectable mannitol (100 ml, IV), tablet phenytoin (100 mg), and injectable dexamethasone (4 mg) were started.

Laboratory investigations revealed a white blood cell count of 12,000 per microliter in complete blood count. Differential leucocyte count showed neutrophil, lymphocyte, monocyte, eosinophil, and basophil percentages of 87.1%, 8.5%, 3.9%, 0.3%, and 0.2%, respectively. In addition, total cell count in the cerebrospinal fluid (CSF) was 500 cells/mm^3^, and differential leucocyte count revealed 80% mononuclear cells. The CSF biochemistry indicated the protein levels of 251 mg/dl and sugar levels of 73 mg/dl. Furthermore, the India ink preparation for CSF was negative.

**Figure 1:**
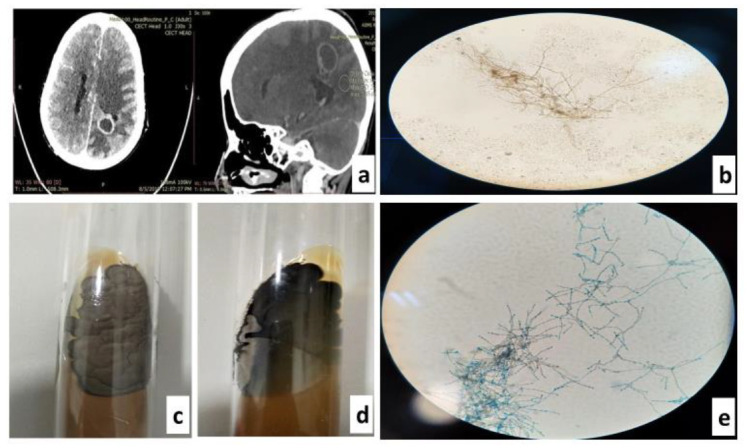
a. Contrast enhanced Magnetic resonance imaging scan revealing a large peripherally enhancing well defined cystic space-occupying axial lesion in left parietal cerebral lobe, with mass effect. b. Pus sample showed numerous pigmented septate hyphae on potassium hydroxide (KOH) mount (Magnification:400x). c. Culture tube showing olivaceous black colonies on obverse. d. Culture tube showing black non-diffusible pigment onreverse. e. Slide culture showing chains of brown, smooth-walled, single-celled, ellipsoid conidia on Lactophenol cotton blue mount (Magnification:400 x).

Contrast-enhanced magnetic resonance imaging scan revealed a large, peripherally enhancing, well- defined, cystic, space-occupying, axial lesion (measuring approximately 33×36×44 mm) in the left parietal lobe with a mass effect (Figure 1a). It showed internal septa and smaller satellite lesions in the surrounding parenchyma. Accordingly, the patient was diagnosed with parietal lobe brain abscess with interventricular rupture.

Surgical intervention was planned, and the patient was subjected to craniotomy. After the radical surgical debridement of necrotic tissue, the pus aspirated from the lesion was sent to the Department of Microbiology for microbiological investigations, including Cepheid's Xpert MTB/RIF assay (Sunnyvale, CA, USA) for tuberculosis, aerobic and anaerobic bacterial culture, and fungal culture. Postoperatively, the GCS score deteriorated to 8/15; therefore, the patient was transferred to the intensive care unit for ventilator support.

On potassium hydroxide mount examination, the pus sample showed numerous pigmented septate hyphae (Figure 1b). Gram staining showed numerous Gram variable hyphae and occasional pus cells; however, no chlamydospores were observed. Culture was performed on selective fungal media, such as Sabouraud dextrose agar and Potato dextrose agar, and then incubated at both 25℃ and 37℃. After 72 h of incubation, the culture tube showed olivaceous black velvety texture colonies on the obverse side and black non-diffusible pigment on the reverse side (figures 1c and 1d).

For the identification of the fungal species, slide culture was performed. Microscopically, the hyphae were brown and septate similar to vegetative hyphae. Long sparsely branched wavy chains of smooth oval conidia that did not display the dark scars of attachment were observed (Figure 1e). A presumptive diagnosis of cerebral phaeohyphomycosis due to *C. bantiana* was established. However, given the lack of facilities at the institute, it was not possible to perform antifungal susceptibility testing on the isolates.

Amphotericin B at a dose of 0.5 mg/kg (IV) once a day and voriconazole at a loading dose of 180 mg, followed by 120 mg (IV), twice a day were added to the treatment. The GCS score improved to 12/15 (E3V4M5) 5 days post-operation, and the vital signs were stable. She was gradually weaned off from the ventilator and shifted to the ward with ongoing antifungal treatment. On day 20 postoperation, she developed acute respiratory distress for which she was again transferred to receive ventilator support. Gradually, she developed multi-organ failure and succumbed to brain abscess caused by *C. bantiana* 27 days after the operation. One of the limitations of the present study was that because of the financial constraints of the patient’s family, no postoperative radiological imaging could be performed to assure the complete surgical resection of infective foci.

**Molecular identification**

The molecular sequencing of the ITS region (ITS1- 5.8S-ITS2) by the National Culture Collection Of Pathogenic Fungi, Post-Graduate Institute of Medical Sciences, Chandigarh, India, conclusively identified the isolate as *C. bantiana* (deposited in the Genbank under the accession number of MT742842).

## Discussion

Phaeohyphomycosis refers to the infections caused by dematiaceous, melanized, or phaeoid fungi, which are a heterogeneous collection of unrelated fungi sharing the common feature of having dihydroxy- naphthalene melanin in the cell walls. They are reported to cause diseases, ranging from superficial infections to life-threatening infections with high mortality, such as brain abscess [ 7 ].

*Cladophialophora bantiana* is a true neurotropic fungus, causing life-threatening CNS fungal infections. Contrary to primary cerebral phaeohyphomycosis caused by other fungi, *C. bantiana* appears to occur more commonly in immunocompetent individuals than in immunocompromised patients [ 8 ]. This species is ubiquitously found in the soil and environment. A majority of these infections are caused by inhalation route leading to subclinical pulmonary infection, followed by hematogenous dissemination to the CNS. However, the pathogenesis of *C. bantiana* phaeohyphomycosis is still uncertain [ 9 ].

In a review study conducted on 234 cases by Chakrabarti et al., the majority (57.3%) of the patients were from Asian countries, especially India (62/124, 50%), and the diagnosis of these cases was delayed with a mean duration of 115 days after entering the symptomatic stage. The disease was equally distributed among immunocompetent and immunosuppressed hosts highlighting the invasive potential of the organism [ 10 ].

Our patient was immunocompetent, and no predisposing factor could be illustrated in her detailed history. She was a housewife from a rural background.

She had some exposures to the soil and vegetative matter in her daily chores of work, which could have exposed her to this fungus. Jon Velasco and Sanjay Revankar stated that an increased risk might be associated with genetic mutations in certain individuals [ 1 ]. *Cladophialophora bantiana* abscesses generally affect the white matter and have a tendency to spread to the frontal lobes [ 11 ]. However, in our patient, the left parietal lobe was affected, which is a rare finding.

The two challenges associated with this fatal infection is early diagnosis and subsequent management. The timely diagnosis of this infection requires a high degree of clinical suspicion. Radiological imaging does not facilitate the differentiation of this infection from other infectious diseases like tuberculosis or other bacterial or fungal causes [ 11 ]. Therefore, the establishment of a prompt and accurate diagnosis requires sample collection by invasive methods, direct microscopy, and culture. It should be also noted that histopathology may provide definitive evidence of phaeohyphomycosis. However, this approach cannot help further in the specific identification of the fungus, which is an essential prerequisite for the targeted management of the patient.

Several studies have shown that radical surgical resection, followed by targeted pharmacological treatment, enables good recovery in many cases [ 6 , 12 , 13 ]. The joint ESCMID/ECMM guidelines for the management of systemic phaeohyphomycosis recommend a complete excision of brain abscesses wherever possible, in combination with antifungal therapy [ 14 ]. The surgical drainage of pus and resection of necrotic tissue paves the way for better penetration of the antifungal agent [ 15 ].

There is no well-defined consensus on the medical management of *C. bantiana cerebral* abscesses. Chakrabarti et al. reported that voriconazole and posaconazole have a good in vitro susceptibility against the fungus. Some other researchers have successfully treated cases using voriconazole, in combination with amphotericin B. In a review study carried out by Revankar et al., the combination of amphotericin B, 5-flucytosine, and itraconazole resulted in improved survival [ 3 , 10 ]. In a study performed in Southern India on two cases of brain abscess caused by *C. bantiana*, the patients were successfully treated with surgical resection and systemic voriconazole therapy [ 15 ]. Although postoperative radiological scan could not be performed, it should be stressed upon since the fungus can infiltrate into the normal-appearing brain tissue, thereby leading to recurrence [ 16 ].

In the absence of comparative trials owing to the rarity of the disease, radical neurosurgical resection, drainage of pus, microbiological identification of the fungi, and prolonged antifungal therapy with close postoperative radiological surveillance are the current treatments of choice for patients with brain abscess caused by *C. bantiana*.

## Conclusion

This case study highlighted that potential diagnostic and therapeutic difficulties can be overcome with the integrated management of the patient by different disciplines of medical science. The differential diagnosis of intraparenchymal fungal abscess should be considered in immunocompetent individuals with ring- enhancing space-occupying lesions. Complete surgical excision and an appropriate antifungal therapy at a proper dose are the key measures for the successful management of otherwise fatal cases.
